# Prevalence of the GFI1-36N SNP in Multiple Myeloma Patients and Its Impact on the Prognosis

**DOI:** 10.3389/fonc.2021.757664

**Published:** 2021-10-25

**Authors:** Cyrus Khandanpour, Christine Eisfeld, Subbaiah Chary Nimmagadda, Marc S. Raab, Niels Weinhold, Anja Seckinger, Dirk Hose, Anna Jauch, Asta Försti, Kari Hemminki, Thomas Hielscher, Manuela Hummel, Georg Lenz, Hartmut Goldschmidt, Stefanie Huhn

**Affiliations:** ^1^ Department of Medicine A, Hematology, Oncology and Pneumology, University Hospital Münster, Münster, Germany; ^2^ Department of Hematology, Oncology and Rheumatology, University Hospital Heidelberg, Heidelberg, Germany; ^3^ Department of Hematology and Immunology, Myeloma Center Brussels & Laboratory for Myeloma research, Vrije Universiteit Brussel (VUB), Jette, Belgium; ^4^ Institute of Human Genetics, University Heidelberg, Heidelberg, Germany; ^5^ Hopp Children’s Cancer Center (KiTZ), Heidelberg, Germany; ^6^ Division of Pediatric Neurooncology, German Cancer Research Center (DKFZ), German Cancer Consortium (DKTK), Heidelberg, Germany; ^7^ Division of Molecular Genetic Epidemiology, German Cancer Research Center (DKFZ), Heidelberg, Germany; ^8^ Division of Cancer Epidemiology, German Cancer Research Center (DKFZ), Heidelberg, Germany; ^9^ Faculty of Medicine and Biomedical Center in Pilsen, Charles University in Prague, Pilsen, Czechia; ^10^ Division of Biostatistics, German Cancer Research Center (DKFZ), Heidelberg, Germany; ^11^ Roche Diagnostics GmbH, Penzberg, Germany; ^12^ National Centre of Tumor Diseases, Heidelberg, Germany

**Keywords:** Gfi1, SNP variant, prevalance, prognosis, multiple myeloma

## Abstract

Transcription factor Growth Factor Independence 1 (GFI1) regulates the expression of genes important for survival, proliferation and differentiation of hematopoietic cells. A single nucleotide polymorphism (SNP) variant of GFI1 (GFI1-36N: serine replaced by asparagine at position 36), has a prevalence of 5-7% among healthy Caucasians and 10-15% in patients with myelodysplastic syndrome (MDS) and acute myeloid leukaemia (AML) predisposing GFI-36N carriers to these diseases. Since GFI1 is implicated in B cell maturation and plasma cell (PC) development, we examined its prevalence in patients with multiple myeloma (MM), a haematological malignancy characterized by expansion of clonal PCs. Strikingly, as in MDS and AML, we found that the GFI1-36N had a higher prevalence among MM patients compared to the controls. In subgroup analyses, GFI1-36N correlates to a shorter overall survival of MM patients characterized by the presence of t(4;14) translocation and gain of 1q21 (≤3 copies). MM patients carrying gain of 1q21 (≥3 copies) demonstrated poor progression free survival. Furthermore, gene expression analysis implicated a role for GFI1-36N in epigenetic regulation and metabolism, potentially promoting the initiation and progression of MM.

## Introduction

GFI1 is a zinc-finger transcriptional repressor with an essential role in controlling hematopoietic stem cell biology, myeloid and lymphoid differentiation and lymphocyte effector functions. The establishment of murine models with constitutive and conditional loss of Gfi1 expression enabled visualization of their cell-specific expression and understanding of Gfi1 function in hematopoietic lineages (1). GFI1 exerts its function as a transcriptional repressor by recruiting histone-modifying enzymes to its target genes (2). GFI1 binds histone deacetylases (HDAC1-3), histone methyltransferases (G9A) or histone demethylases (LSD1) and recruits them to their target genes. In a stepwise process, it induces deacetylation of lysine 9 of histone 3 (H3K9) followed by dimethylation of H3K9 or de-methylation of histone 3, lysine 4 (H3K4), resulting in gene silencing (1).

We previously reported that a coding single nucleotide polymorphism (SNP) in the human GFI1 (rs34631763, denominated as *GFI1-36N*) predisposes carriers to myelodysplastic syndrome (MDS) and acute myeloid leukaemia (AML) and influenced their prognosis (3, 4). On the molecular level, the GFI1-36N protein differs from the more common GFI1-36S with regards to its ability in inducing epigenetic changes as deacetylation of H3K9 at the HOXA9 locus (3, 4). However, genome-wide H3K9-acetylation level of GFI1 target genes was increased in hematopoietic progenitor cells of GFI1-36N mice and primary murine and human GFI1-36N leukemic cells (3). Higher H3K9-acetylation of the genes in GFI1-36N-expressing cells correlated with higher expression and activation of genes facilitating AML development (3).

Several publications previously reported the association of at least 24 independent loci carrying germline variants associated with increased risk of development of multiple myeloma (MM) (2, 5–8). MM is a B cell malignancy characterized by a multistep accumulation of genetic and epigenetic changes leading to malignant transformation and proliferation of plasma cells (PCs) (9, 10). MM prognosis depends on age, stage, overall performance status and chromosomal aberrations and gene mutations (10). Since GFI1 plays an important role in B-cell development and subsequent PC differentiation (11, 12) we investigated whether the presence of GFI1-36N might predispose carriers to MM and affect their prognosis. Several genetic aberrations are strongly associated with MM treatment response and patient survival (13). Of these, translocation (4, 14) and gain of 1q21 are associated with poor prognosis (13, 14). In this study, we investigated the frequency of the germline *GFI1-36N* and its impact on overall survival (OS) and progression-free survival (PFS) of MM patients. We finally investigated how the GFI1-36N SNP variant potentially altered the overall gene expression pattern of GFI1-36N homo or heterozygous PCs.

## Materials and Methods

### Patients

We determined the frequency of germline *GFI1-36N* homo- or heterozygous carriers among a cohort of 1229 newly diagnosed MM patients and 2005 unaffected control persons based on published genome-wide association study (GWAS) data of patients treated within the German-Speaking Myeloma Multicenter Group (GMMG), HD3, HD4 and MM5 trials. The characteristics of the patient and control groups have been described earlier (2, 6, 15).

### Gene Expression Analysis

Gene expression profiling using U133 2.0 plus arrays (Affymetrix, Santa Clara, CA, USA) was performed as published in MM patients (n=716, 637 were homozygous for *GFI1-36S*, 79 hetero- or homozygous for *GFI1-36N*) (16). Gene set enrichment analysis of Gene Ontology pathways between *GFI1-36S* and *GFI1-36N* was performed as published before (17). The analysed data-set have been published at the following link: https://www.ebi.ac.uk/arrayexpress/experiments/E-MTAB-2299/.

### Statistics

Fisher’s exact test and Wilcoxon test were used to assess the association of the genotypes with categorical and continuous parameters, respectively. Logistic regression was used to estimate the odds ratio and corresponding 95% confidence interval. No adjustment for multiples testing was required since the GFI1-36N locus was selected a priori. Cox regression and log-rank test were used to assess the prognostic impact. Kaplan-Meier estimates were used to estimate distribution of PFS and OS times.

## Results

### Prevalence of the GFI1-36N Variant Allele and Its Association With Key Characteristics of MM Patients

**Figure 1:**
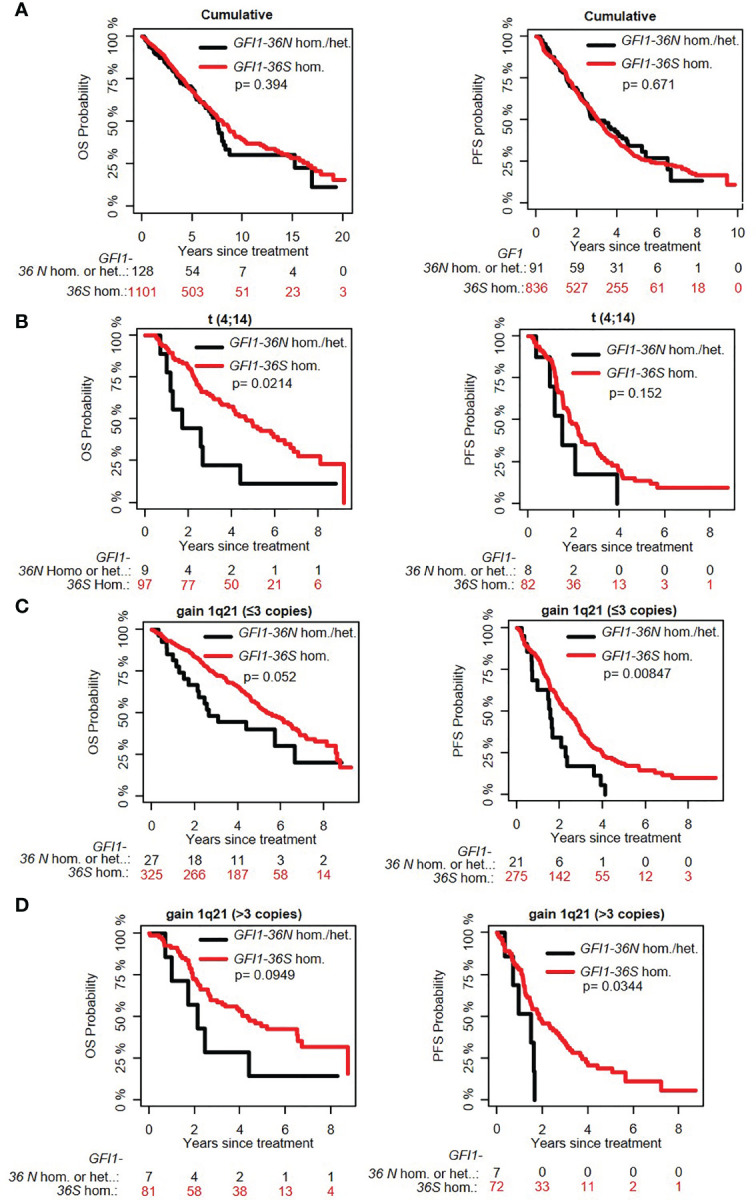
Influence of the presence of GFI1-36N allele on PFS and OS of MM patient subgroups. **(A)** GFI1-36N did not influence OS and PFS in the entire cohort. **(B)** GFI1-36N negatively affects OS but not PFS in MM patients with t(4;14) translocation. **(C, D)** GFI1-36N negatively affects OS and PFS in MM patients with gain of 1q21 (≤3 copies and >3 copies).

**Figure 2:**
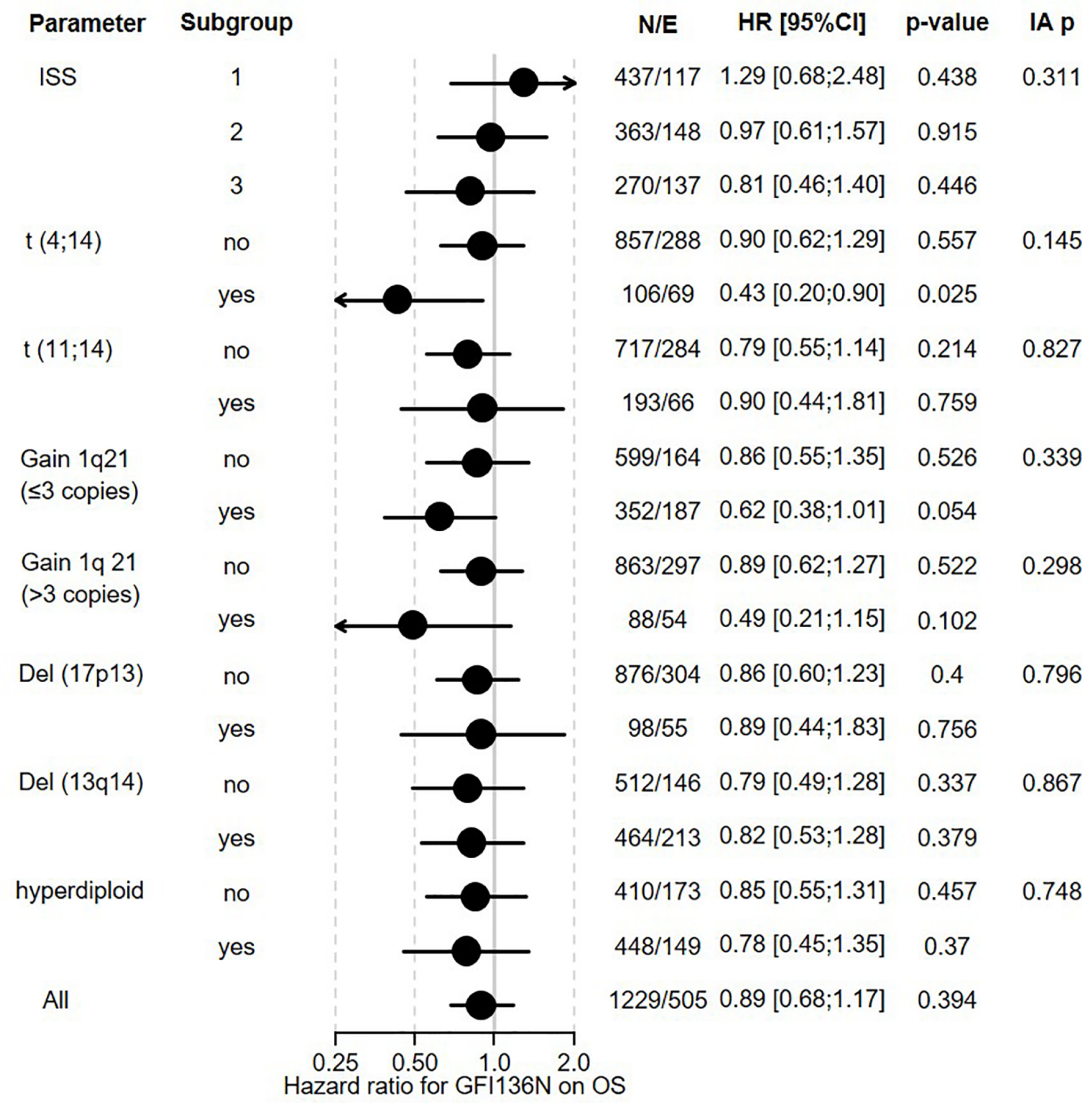
Influence of GFI1-36N on OS of MM patients. GFI1-36N (homo or heterozygous) MM patients were stratified according to presence/absence/levels of different parameters, International Staging System (ISS), t(4;14), t(11;14), gain 1q21 (≤3 copies), or gain 1q21 (>3 copies), Del (17p13), Del (13q14), hyperdiploid and statistically evaluated for PFS. Hazard ratio including 95% confidence interval based on Cox regression is presented. IA p indicates test on the interaction between subgroups, N/E: Number of patients and events within the subgroup.

**Figure 3:**
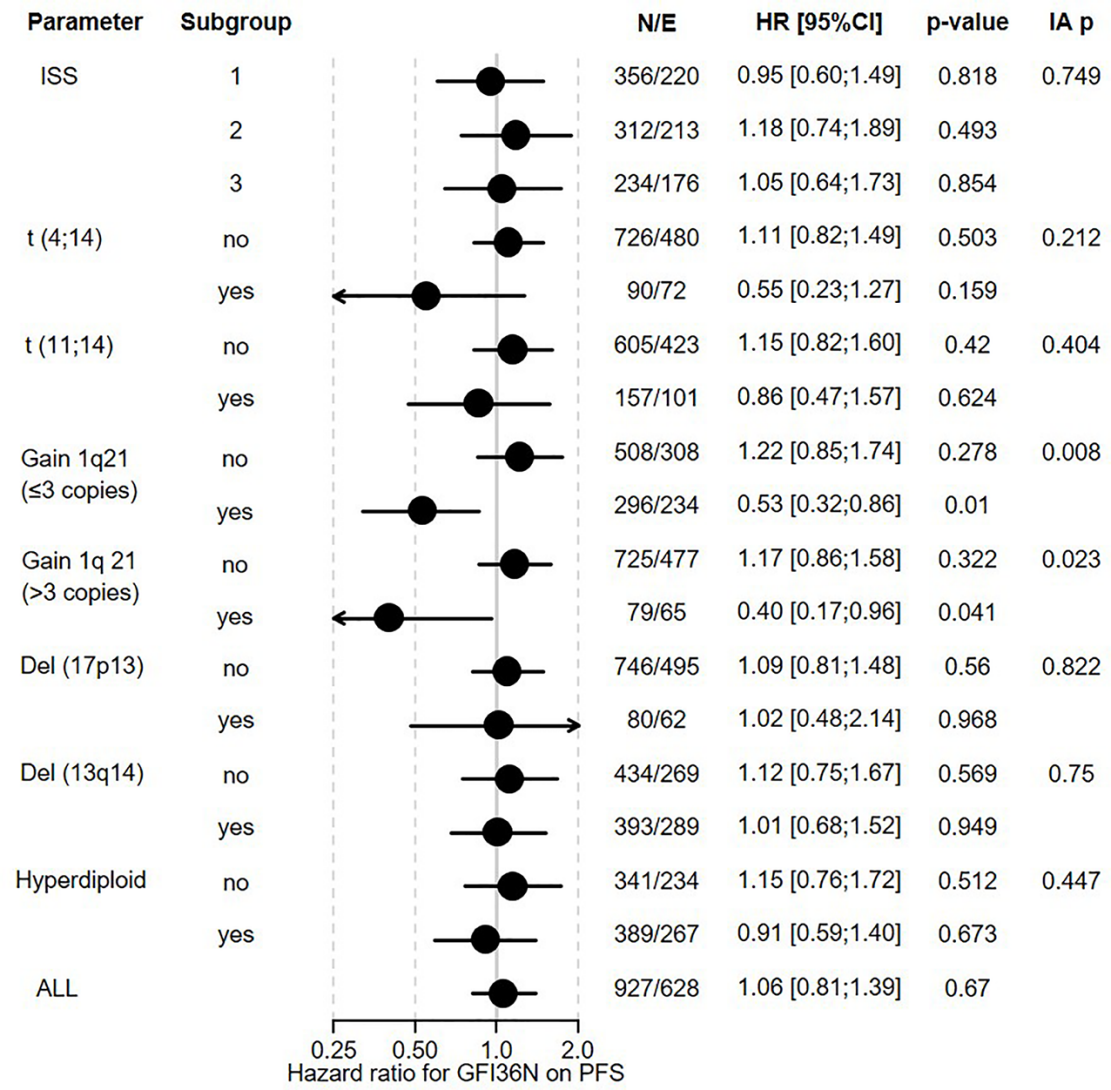
Influence of GFI1-36N on PFS of different subgroups of MM patients. GFI1-36N (homo or heterozygous) MM patients were stratified according to presence/absence/levels of different parameters, International Staging System (ISS), t(4;14), t(11;14), gain 1q21 (≤3 copies), or gain 1q21 (>3 copies), Del (17p13), Del (13q14), hyperdiploid and statistically evaluated for PFS. Hazard ratio including 95% confidence interval based on Cox regression is presented. IA p: test on the interaction between subgroups, N/E: Number of patients and events within the subgroup.

The overall prevalence of the *GFI1-36N* allele was 7.9% among healthy controls and 10.4% among MM patients indicating an association of the GFI1-36N allele with the risk of developing MM (OR 1.35, 95%CI 1.06-1.72, p-value 0.016; corrected for population stratification; **Table 1**). There was no significant difference between *GFI1-36S* homozygous and *GFI1-36N* homo-or heterozygous MM patients concerning age, sex, ISS stage or GFI1-RNA expression level (**Tables 2**, **3**). To evaluate the effect of the GFI1-36N allele on disease progression, overall survival (OS) and progression-free survival (PFS) of MM patients treated within the MM5 trial was examined (**Figure 1A**). The MM5 phase III trial examined the impact of induction therapy with doxorubicin, bortezomib and dexamethasone versus cyclophosphamide in combination with bortezomib and dexamethasone (15). Log-rank test and Cox regression were used to compare PFS and OS between groups. The presence of the *GFI1-36N* allele did not influence OS and PFS (**Figures 1A**, **2** and **3**). Of note, in a subset of MM patients characterized by the presence of t(4,14) translocation, *GFI1-36N* demonstrated a negative impact on OS (Log-rank: p= 0.02) but not on PFS (**Figures 1B**, **2** and **3**). Furthermore, in MM patients characterized by the gain of 1q21 (≤3 copies), GFI1-36N demonstrated a negative impact with a borderline statistical significance on OS and with significance on PFS (Log-rank: p= 0.052 and 0.008, respectively (**Figures 1C**, **2** and **3**). Of note, Gain of 1q21 (>3 copies) was associated with negative PFS (p=0.034, **Figures 1D**, **2** and **3**). It has been previously shown that gain of 1q21 involves genes such as *BCL9, MCL1, CKS1B* and *ANP32E*, which contribute either to inhibition of apoptosis or enhancement of cell cycling or epigenetic modification (18, 19).

**Table 1:** The frequency of the GFI1-36N allele was determined within a population of newly diagnosed MM patients and a respective control population. OR 1.35, 95%CI 1.06-1.72.

	Controls (n = 2005)		MM cases (n = 1229)		p-value
	n	%	n	%	
GFI1-36N allele homozygous	5	0.2	2	0.2	
GFI1-36N allele heterozygous	154	7.7	126	10.2	
GFI1-36N allele homozygous + heterozygous	159	7.9	128	10.4	P=0.02
GFI1-36S allele homozygous	1846	92.1	1101	89.6	

**Table 2:** Clinical factors and their association with GFI136N. No significant association between the presence of the GFI1-36N allele and gender or ISS was observed.

	*GFI1-36N Homo or heterozygous*					
Prognostic factor	n	%	n	%	p-value	OR
						95%CI
**Gender**					0.51	0.88 (0.621-1.28
**Male**	72	56.2	653	59.3		
**Female**	56	43.8	448	40.7		
**ISS stage**					0.37	
**I**	40	36.4	397	41.4		
**II**	44	40	319	33.2		(II vs I) 1.37
						(0.87-2.16)
**III**	26	23.6	244	25.4		(III vs I) 1.06
						(0.62-1.77)

**Table 3:** Correlation between presence of GFI1-36N allele and age or GFI1-RNA expression level.

Variable	Group	n	Min	Q1	Median	Mean	Q3	Max
Age	*GFI1-36N* homo or heterozygous	128	37	51	57	56.4	62.5	70
P=0.33	*GFI1-36S* homozygous	1101	24.8	52	58	56.9	63	73.4
OR (per 10 year) 0.93 (0.75-1.16)	All	1229	24.8	51.8	58	56.8	63	73.4
*GFI1* expression	*GFI1-36N* homo or heterozygous	79	3.4	7.6	8.3	8	8.8	10.2
P=0.71								
OR (per FC increase)								
1.02 (0.87-1.22)								
	*GFI1-36S* homozygous	637	3.4	7.4	8.2	8	8.9	11.1
	All	716	3.4	7.4	8.2	8	8.9	11.1

No significant difference was seen between GFI1-36N homo or heterozygous MM patients on one hand and GFI1-36S homozygous patients on the other hand concerning age or GFI1expression. FC, Fold change.

We next determined potential pathways by which presence of GFI1-36N might alter gene expression pattern in PC. Analysing the gene expression profile between *GFI1-36S* and *-36N* patient groups, we found that pathways responsible for epigenetic regulation were upregulated and those regulating metabolism were down-regulated in plasma cells of heterozygous *GFI1-36S* and homozygous *-36N* MM patients (**Table 4**). This is again in line with earlier reports that GFI1 is implicated in metabolic regulation and this might contribute to the malignant transformation (20). This corresponds to our previous observations in GFI1-36N myeloid malignancies, whereby GFI1-36N failed to induce epigenetic changes to the same extent as the GFI1-36S protein (3, 21).

**Table 4:** Changes in gene expression of *GFI1-36N* homo- or heterozygous myeloma cells as compared to *GFI1-36S* homozygous cells.

Pathway	Genes (n)	Trend	p Value	FDR
Ras guanyl-nucleotide exchange factor activity	87	Up	9,44E-06	0,001987
DNA-binding transcription activator activity, RNA polymerase II-specific	230	Up	2,04E-05	0,003533
Histone demethylase activity	17	Up	2,74E-05	0,004463
Rho guanyl-nucleotide exchange factor activity	45	Up	3,1E-05	0,004977
RNA polymerase II regulatory region DNA binding	383	Up	3,52E-05	0,005439
RNA polymerase II regulatory region sequence-specific DNA binding	381	Up	4,49E-05	0,006491
Regulatory region nucleic acid binding	486	Up	5,98E-05	0,008347
Transcription regulatory region sequence-specific DNA binding	413	Up	6,11E-05	0,008423
Transcription regulatory region DNA binding	485	Up	6,35E-05	0,008459
Sequence-specific DNA binding	557	Up	6,82E-05	0,008957
Sequence-specific double-stranded DNA binding	438	Up	6,89E-05	0,008957
Mitochondrial respiratory chain	63	Down	2,44E-14	2,83E-10
Oxidative phosphorylation	103	Down	1,36E-13	7,88E-10
Respiratory chain complex	55	Down	2,85E-13	8,61E-10
ATP synthesis coupled electron transport	70	Down	4,51E-13	1,05E-09
Mitochondrial protein complex	203	Down	6,36E-13	1,09E-09
Mitochondrial ATP synthesis coupled electron transport	69	Down	6,6E-13	1,09E-09
Inner mitochondrial membrane protein complex	94	Down	9,55E-13	1,38E-09
Respiratory electron transport chain	84	Down	1,44E-12	1,86E-09
Mitochondrial inner membrane	367	Down	9,1E-12	1,05E-08
Mitochondrial respiratory chain	63	Down	2,44E-14	2,83E-10
Oxidative phosphorylation	103	Down	1,36E-13	7,88E-10

FDR, False discovery rate.

## Discussion

Our previous investigations and observations have underscored a role for *GFI1*-36S and -36N SNP variants in myeloid malignancies. We had reported that the presence of the GFI1-36N protein was associated with an increased incidence of mutations in genes encoding epigenetic modifiers such as *DNMT3a* and could be therapeutically exploited in AML therapy (2, 15). One of the physiological functions of GFI1 is to recruit histone-modifying genes to its target genes and induce repressive epigenetic changes. GFI1 also regulates lymphoid development in general and B-cell development in particular. Hence it would be conceivable that the presence of GFI1-36N might not only disturb myeloid development but also B-cell development and predispose to myeloma development. Therefore, we evaluated the prevalence of the GFI1-36N variant and if it contributed to the pathogenesis of MM. Interestingly, our results are in line with observations for AML. They indicate that GFI1-36N has a higher prevalence among MM patients compared to the unaffected population. Prevalence among control persons was slightly higher compared to our previous studies, which reported frequencies of *GFI1-36N* allele between 3-7% in the different control groups (4, 21). The frequency of the *GFI1-36N* allele among MM patients was similar to our previous reports in MDS and AML patients varying between 10-15% with an OR of 1.3-2 (4, 21). A similar frequency of the *GFI1-36N* allele among AML, MDS and MM patients points to a possible universal role of GFI1-36N predisposing or contributing to haematological malignancies. Our results with global gene expression pattern indicate that a similar mechanism might also explain the pathogenesis and therefore indicate that GFI1-36N appears to influence the pathogenesis of MM. It would therefore be well conceivable that the presence of a GFI1-36N protein prepares an epigenetic landscape for malignant transformation and mutation accumulation involving t(4,14) translocation, gain of 1q21 and thereby might contribute to an evolution of tumour cells. It remains to be elucidated whether our findings with an elevated frequency of GFI1-36N in myeloma patients and its potential influence on the disease course of t(14;16) and gain 1q21, can be replicated in other independent cohorts. However it could be that GFI1-36N is a general factor predisposing to development of myeloid malignancies and myeloma.

## Data Availability Statement

The analysed data-set have been published at the following link: https://www.ebi.ac.uk/arrayexpress/experiments/E-MTAB-2299/.

## Author Contributions

CK, CE, SN, MR, NW, AS, DH, AJ, AF, KH, TH, MH, GL, HG, and SH provided and analysed the data. CK and SH designed the study and wrote the manuscript. All authors contributed to the article and approved the submitted version.

## Funding

DH was supported by Deutsche Forschungsgemeinschaft (SFB/TRR79) the German Federal Ministry of Education (“CLIOMMICS” (01ZX1309 and 01ZX1609) as well as “CAMPSIMM” (01ES1103)). KH is supported by the European Union’s Horizon 2020 research and innovation programme, grant No 856620. CK is supported by the Jose Carreras Leukaemia Foundation (DJCLS 17R/2018), partially by the Deutsche Krebshilfe (70112392), Deutsche Forschungsgemeinschaft (KH331/2-3), and the intramural funding of the Faculty of Medicine at University Hospital of Muenster (Kha2/002/20).

## Conflict of Interest

Author MH was employed by company Roche Diagnostics GmbH.

The remaining authors declare that the research was conducted in the absence of any commercial or financial relationships that could be construed as a potential conflict of interest.

## Publisher’s Note

All claims expressed in this article are solely those of the authors and do not necessarily represent those of their affiliated organizations, or those of the publisher, the editors and the reviewers. Any product that may be evaluated in this article, or claim that may be made by its manufacturer, is not guaranteed or endorsed by the publisher.
